# Anti-PDHA1 antibody is detected in a subset of patients with schizophrenia

**DOI:** 10.1038/s41598-020-63776-0

**Published:** 2020-05-13

**Authors:** Yukako Nakagami, Genichi Sugihara, Noriyuki Nakashima, Masaaki Hazama, Shuraku Son, Shuhe Ma, Riki Matsumoto, Toshiya Murai, Akio Ikeda, Kosaku Murakami

**Affiliations:** 1 Department of Psychiatry, Kyoto University Graduate School of Medicine, Kyoto, Japan; 2 Kyoto University Health Service, Kyoto, Japan; 3 Department of Physiology, Kurume University School of Medicine, Kurume, Japan; 4 Department of Rheumatology and Clinical Immunology, Kyoto University Graduate School of Medicine, Kyoto, Japan; 5 Division of Neurology, Kobe University Graduate School of Medicine, Kobe, Japan; 6 Department of Epilepsy, Movement Disorders and Physiology, Kyoto University Graduate School of Medicine, Kyoto, Japan; 7 Department of Psychiatry and Behavioral Sciences, Graduate School of Medical and Dental Sciences, Tokyo Medical and Dental University, Tokyo, Japan

**Keywords:** Neuroimmunology, Neuroscience

## Abstract

Autoantibodies have been implicated in schizophrenia aetiology. Here, novel autoantibodies were isolated from patients with schizophrenia. Autoantibody candidates were searched using two-dimensional gel electrophoresis and western blotting with rat brain proteins as antigens and two sera pools (25 schizophrenia patients versus 25 controls) as antibodies. Immunoreactive antigens were identified by mass spectrometry. Antibody prevalence were evaluated by western blotting using human recombinant proteins. Furthermore, brain magnetic resonance imaging data (regional brain volumes and diffusion tensor imaging measures) were compared. Two proteins of the mitochondrial respiration pathway were identified as candidate antigens. Three patients with schizophrenia, but no controls, expressed antibodies targeting one of the candidate antigens, i.e., pyruvate dehydrogenase E1 component subunit alpha, somatic form, mitochondrial (PDHA1, EC 1.2.4.1), which is related to mitochondrial energy production. Anti-PDHA1 antibody-positive patients (n = 3) had increased volumes in the left occipital fusiform gyrus compared to both controls (n = 23, p = 0.017) and antibody-negative patients (n = 16, p = 0.009), as well as in the left cuneus compared to antibody-negative patients (n = 16, p = 0.018). This is the first report of an anti-PDHA1 antibody in patients with schizophrenia. Compatible with recent findings of mitochondrial dysfunction in schizophrenia, this antibody may be involved in the pathogenesis of a specific subgroup of schizophrenia.

## Introduction

Schizophrenia is a mental disorder characterised by hallucinations, delusions, and cognitive dysfunction. Schizophrenia typically develops in early adulthood^1^; the first episode of psychosis is often preceded by prodromal symptoms such as cognitive dysfunction^1^. The lifetime prevalence of schizophrenia is approximately 1% worldwide^2^. The diagnosis of schizophrenia is mainly based on subjective psychiatric symptoms and lacks any objective signs including diagnostic markers.

The pathophysiology of schizophrenia is heterogeneous, and various underlying mechanisms have been reported^1^. One of the most promising mechanisms is immune dysfunction. This assumption is supported by genetic, epidemiological, and post-mortem brain studies^1–4^. It is also corroborated by research investigating the effects of antipsychotic agents. Studies have indicated that antipsychotic agents can modulate the immune system in addition to the nervous system^2^.

Anti-neuronal antibodies have attracted considerable attention in research related to immune dysfunction in schizophrenia. In 2007, an antibody targeting N-methyl-D-aspartate (NMDA) receptors, one of the glutamate receptors, was reported to cause autoimmune encephalitis manifesting with schizophrenia-like symptoms^5,6^. Despite their similarities in psychotic symptoms, the treatment strategy of anti-NMDA-receptor antibody encephalitis differs from that of schizophrenia. The former can be cured by immunotherapy (e.g., glucocorticoids and plasma exchange) and/or tumour removal (in paraneoplastic syndromes), whereas the latter can only be treated symptomatically with psychotropic agents^1,6^. Although it is important to assess the presence of anti-NMDA antibodies in patients with schizophrenia from the perspective of therapeutic strategy, recent studies have demonstrated the absence or minimal prevalence of anti-NMDA-receptor antibody-positive cases in patients with schizophrenia^7,8^.

**Figure 1:**
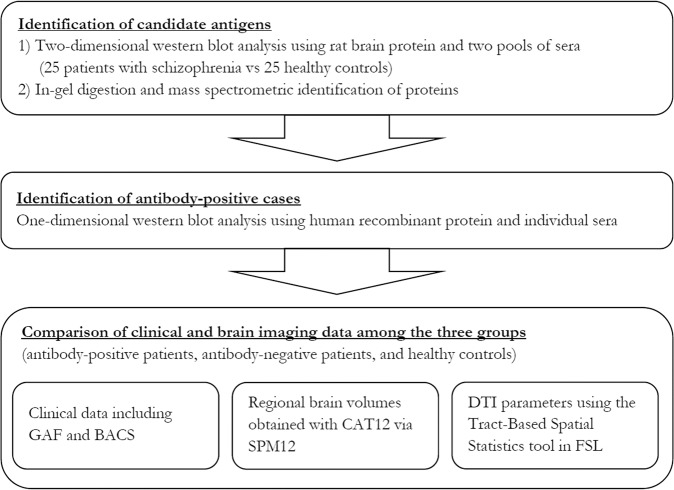
Flow chart of the experimental method. BACS, Brief Assessment of Cognition in Schizophrenia; CAT, Computational Anatomy Toolbox; DTI, diffusion tensor imaging; GAF, Global Assessment of Functioning scales; SPM, Statistical Parametric Mapping Software.

Considering the numerous studies supporting an immune dysfunction in schizophrenia, anti-NMDA-receptor antibodies may not be the only antibodies providing an association between schizophrenia and autoimmune dysfunction^2–4^. The possibility remains that other, unidentified antibodies can also induce psychotic symptoms in people currently diagnosed with schizophrenia. However, the evaluation of a wide spectrum of known autoantibodies did not reveal antibodies related to schizophrenia^9^. Thus, unknown or novel antibodies should be identified using a comprehensive proteomic analysis. In the current study, we searched for novel autoantibodies against brain tissue in sera of patients with schizophrenia and healthy controls using two-dimensional gel electrophoresis-based proteomic analysis^10^. We then evaluated the prevalence of the detected antibodies and assessed their characteristics using psychiatric tests and brain magnetic resonance imaging (MRI) data. The general approach of this study is shown in Fig. 1.

## Results

### Study participants

Table 1 shows the demographic and clinical characteristics of the participants. Consistent with previous studies^11,12^, the schizophrenia patient group showed significantly lower scores in estimated premorbid intelligence quotient (IQ) and Global Assessment of Functioning scales (GAF), as well as in several domains of the Brief Assessment of Cognition in Schizophrenia (BACS) and quality of life (QOL)-26.

**Table 1:** Demographic and clinical characteristics of the study participants.

	Healthy controls					Schizophrenia					p-values
	Average n (%)	Minimum	Maximum	SD	n	Average n (%)	Minimum	Maximum	SD	n	
Sex (male)	14 (56%)				25	14 (56%)				25	1.00
Age	44.3	20	60	12.1	25	44.8	20	69	13.6	25	0.79
Current smokers	3 (12.5%)				24	5 (25%)				20	0.43
Education (years)	14.7	12	16	1.6	24	13.6	9	21	2.7	19	0.07
Estimated premorbid IQ	108.5	98	120	5.8	25	101.2	85	118	9.4	25	0.002**
Age of onset						26.2	12	59	10.1	25	
Duration of illness (year)						18.6	1	50	11.7	25	
Duration of untreated illness (years)						0.9	0	12	2.6	25	
**Medication**											
CPZ eqv.(mg/day)						519.3	0	1466.7	365.1	20	
-SGA dosage (mg/day CPZ eqv.)						486.0	0	1206.1	302.0	20	
-FGA dosage (mg/day CPZ eqv.)						33.3	0	666.7	145.3	20	
Biperiden eqv. (mg/day)						0.7	0	4	1.2	20	
Imipramine eqv. (mg/day)						23.4	0	187.5	53.3	20	
Diazepam eqv. (mg/day)						5.2	0	30	7.4	20	
**PANSS total**						63.2	32	100	19.1	24	
-Positive scale						13.9	7	27	5.4	24	
-Negative scale						18.1	7	28	5.6	24	
-General psychopathology scale						31.1	17	50	9.9	24	
**GAF**											
-GAF-symptom	87.4	75	100	8.2	22	63.0	35	90	14.3	24	<0.001**
-GAF-function	86.3	75	100	7.2	22	57.8	35	80	14.5	24	<0.001**
**BACS, Z-scores**											
-List Learning	0.1	−7.3	3.4	1.7	25	−1.4	−4.8	2.3	1.8	19	0.001**
-Digit Sequencing Task	0.2	−6.7	2.3	1.8	25	−0.4	−2.9	1.9	1.3	19	0.26
-Token Motor Task	−0.1	−6.5	1.5	1.6	25	−1.3	−2.9	1.3	1.1	19	0.002**
-Symbol Coding	0.0	−6.1	1.8	1.5	25	−1.5	−4.8	0.8	1.2	19	0.001**
-Verbal Fluency	0.3	−6.5	3.0	1.8	25	−0.8	−3.8	2.7	1.5	19	0.03*
-Tower of London Test	−0.4	−10.6	1.6	2.2	25	−0.6	−3.9	1.4	1.4	19	0.73
**QOL-26**											
-Physical domain	3.7	2.6	5.0	0.7	25	2.8	1.7	4.0	0.7	19	<0.001**
-Psychological domain	3.4	1.5	5.0	0.8	25	2.7	1.3	3.8	0.6	19	0.003**
-Social relationships	3.2	1.7	4.3	0.7	25	2.8	1.0	4.3	0.9	19	0.24
-Environment	3.5	2.1	4.5	0.6	25	3.3	1.8	4.1	0.5	19	0.44
-General health	3.4	1.0	5.0	1.0	25	2.4	1.0	4.5	1.1	19	0.004**

BACS, Brief Assessment of Cognition in Schizophrenia; CPZ, chlorpromazine; eqv., equivalent; FGA, first-generation antipsychotic; GAF, Global Assessment of Functioning scales; IQ, intelligence quotient; PANSS, Positive and Negative Syndrome Scale; QOL, quality of life; SD, standard deviation; SGA, second-generation antipsychotic

Categorical and continuous variables were assessed with Fisher’s exact test and the t-test (or Mann–Whitney U test), respectively. **p < 0.01, *p < 0.05.

### Identification of target proteins

**Figure 2:**
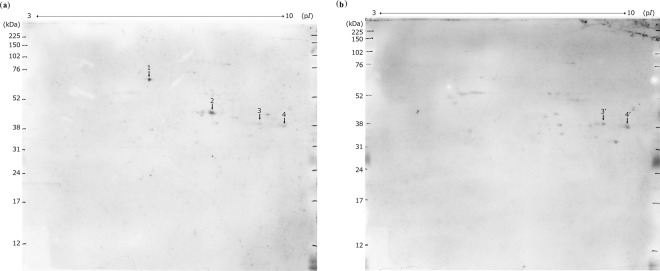
Two-dimensional western blotting of rat brain proteins. Representative images showing the two-dimensional gel electrophoresis followed by western blot in duplicate experiments. Two pools of sera from patients with schizophrenia (n = 25, 100 μg rat brain protein, **a**) and healthy controls (n = 25, 100 μg rat brain protein, **b**) were used at 1:1,600 dilution. As the secondary antibody, a peroxidase-labelled antibody against human IgA + IgG + IgM was used at 1:400,000 dilution. (**a**) The four arrows indicate the protein spots that reacted with pooled sera from 25 patients with schizophrenia. Among these spots, numbers 1 and 2 indicate protein spots that reacted with sera from patients with schizophrenia but not with those from healthy controls. (**b**) The two arrows (spot numbers 3′and 4′) indicate protein spots that reacted with both pools, i.e., sera from patients with schizophrenia and healthy controls. kDa, kilodalton; p*I*, isoelectric point.

Figure 2 and Supplementary Fig. S1 show two-dimensional western blots of rat brain proteins. Consistently, two protein spots demonstrated an exclusive reaction with sera obtained from patients with schizophrenia and were subsequently analysed using matrix-assisted laser desorption/ionisation time-of-flight (MALDI-TOF/TOF).

Table 2 shows the identity of these proteins: dihydrolipoyllysine-residue acetyltransferase component of pyruvate dehydrogenase complex, mitochondrial (DLAT, EC 2.3.1.12; gi number 78365255) and pyruvate dehydrogenase E1 component subunit alpha, somatic form, mitochondrial (PDHA1, EC 1.2.4.1; gi number 124430510). Both candidate antigens were proteins related to the mitochondrial respiration pathway. Based on these findings, we tested additionally the presence of anti-mitochondrial antibodies used in the diagnosis of primary biliary cholangitis with a sensitivity of 90.5% and a specificity of 94.0%^13^; however, primary biliary cholangitis was not concomitant in all the participants.

**Table 2:** Proteins in the two-dimensional gel (Fig. 2) identified by mass spectrometry and database searches.

Spot in Fig. 2	Accession No.	Protein	Sequence coverage (%)	MOWSE Score
1	gi|78365255	Dihydrolipoyllysine-residue acetyltransferase component of pyruvate dehydrogenase complex, mitochondrial (EC 2.3.1.12)	18	359
2	gi|124430510	Pyruvate dehydrogenase E1 component subunit alpha, somatic form, mitochondrial (EC 1.2.4.1)	29	381

EC, enzyme commission number; MOWSE, molecular weight search.

### One-dimensional western blot analysis using human recombinant protein and individual sera

**Figure 3:**
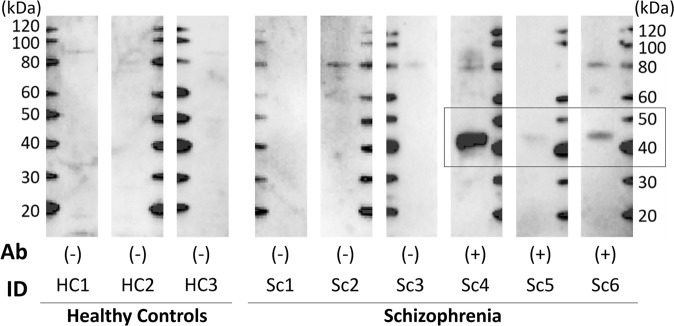
One-dimensional western blotting of human recombinant protein using individual sera. Results of one-dimensional gel electrophoresis using the human recombinant protein PDHA1 (42 kDa,300 ng per lane) followed by western blotting are shown. Individual sera were used at 1:200 dilution, and a peroxidase-labelled secondary antibody against human IgA + IgG + IgM was used at 1:30,000 dilution. Three patients with schizophrenia (Sc4-Sc6) had specific positive signals (black rectangle). As examples of negative signals, images of both healthy controls (HC1-HC3) and patients with schizophrenia without specific signals (Sc1-Sc3) are included. The full original blots are shown in Supplementary Fig. S2.

The one-dimensional western blot analysis using PDHA1 exhibited specific positive signals in 3 of 25 patients with schizophrenia (named Sc4, Sc5, and Sc6 in Fig. 3). These positive signals that were not found in any of the 25 healthy controls differed in strength (i.e., Sc4 > Sc6 > Sc5; see Fig. 3) and were reproducible. These differences in immunoreactivity may be attributable to individual levels of antibody titres. The corresponding DLAT analysis revealed no positive signals among patients with schizophrenia or healthy controls.

### Clinical characteristics of the identified antibody-positive patients

Table 3 shows the clinical data of the antibody-positive cases. Their average age was 43.0 years compared to 44.3 and 45.0 years in healthy controls and antibody-negative schizophrenia, respectively. The average disease duration in antibody-positive schizophrenia was 18.7 years, whereas that in antibody-negative schizophrenia was 18.6 years. Thus, in the following analysis, corrections for age and disease duration were not employed. Compared to the data in Table 1, no unique, distinguishing characteristic was found in antibody-positive cases, although a definitive conclusion is precluded because of the small number (n = 3). Differences in western blot reactivity (i.e., Sc4 > Sc6 > Sc5; Fig. 3) were not obvious, either.

**Table 3:** Demographic and clinical characteristics of anti-PDHA1 antibody-positive patients with schizophrenia.

ID in Fig. 3	Sc4	Sc5	Sc6
Sex	Male	Male	Male
Age	55	41	33
Current smokers	Non-smoker	Non-smoker	Non-smoker
Education (years)	14	16	10
Estimated premorbid IQ	98	114	108
Age of onset	28	31	14
Duration of illness (year)	27	10	19
Duration of untreated psychosis (years)	0	0	0
Medication	Risperidone 2 mg Aripiprazole 18 mg Flunitrazepam 2 mg Ramelteon 8 mg Clonazepam 1 mg Biperiden Hydrochloride 2 mg	Olanzapine 12.5 mg	Paroxetine 25 mg Risperidone 1.5 mg
PANSS total	100	43	56
-Positive scale	25	10	10
-Negative scale	25	15	20
-General psychopathology scale	50	18	26
**GAF**			
-GAF-symptom	51	75	71
-GAF-function	41	65	71
**BACS, Z-scores**			
-List Learning	0.5	−2.8	2.3
-Digit Sequencing Task	1.9	1.5	0.2
-Token Motor Task	−1.8	0.1	−2.9
-Symbol Coding	−0.6	−1.4	−1.3
-Verbal Fluency	0.0	2.7	1.0
-Tower of London Test	0.4	−2.3	−1.3
**QOL-26**			
-Physical domain	2.1	3.0	1.7
-Psychological domain	2.2	2.8	1.3
-Social relationships	2.3	2.7	2.3
-Environment	3.3	3.8	3.5
-General health	2.0	1.0	1.0

BACS, Brief Assessment of Cognition in Schizophrenia; GAF, Global Assessment of Functioning scales; IQ, intelligence quotient; PANSS, Positive and Negative Syndrome Scale; QOL, quality of life.

### Brain imaging characteristics of the antibody-positive patients

**Figure 4:**
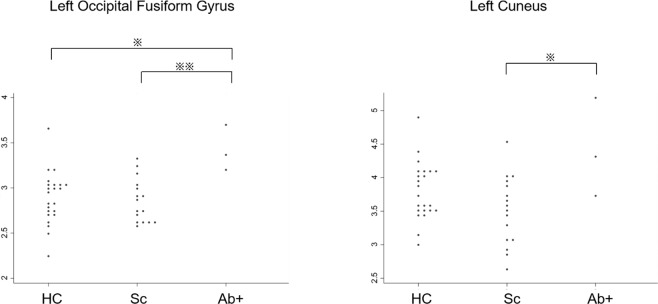
MRI brain volumes displaying significant differences in antibody-positive patients with schizophrenia. Regional brain volumes are compared between healthy controls (HC), antibody-negative patients with schizophrenia (Sc), and antibody-positive patients with schizophrenia (Ab+). Only regions with statistical differences in Ab + are shown in these dot plot graphs. Statistical significance was determined at p-values < 0.01 (**) and <0.05 (*) using the Kruskal–Wallis test followed by Dunn’s post hoc test.

All available MRI data, except for the data from three patients who underwent MRI scans using a different machine, were used in our analysis including 23 healthy controls, 16 antibody-negative patients, and 3 antibody-positive patients. Anti-PDHA1 antibody-positive patients with schizophrenia had increased volumes of the left occipital fusiform gyrus compared to both healthy controls (p = 0.017) and antibody-negative patients (p = 0.009; Fig. 4). We also found increased volumes of the left cuneus in antibody-positive patients compared to antibody-negative patients (p = 0.018; Fig. 4).

**Figure 5:**
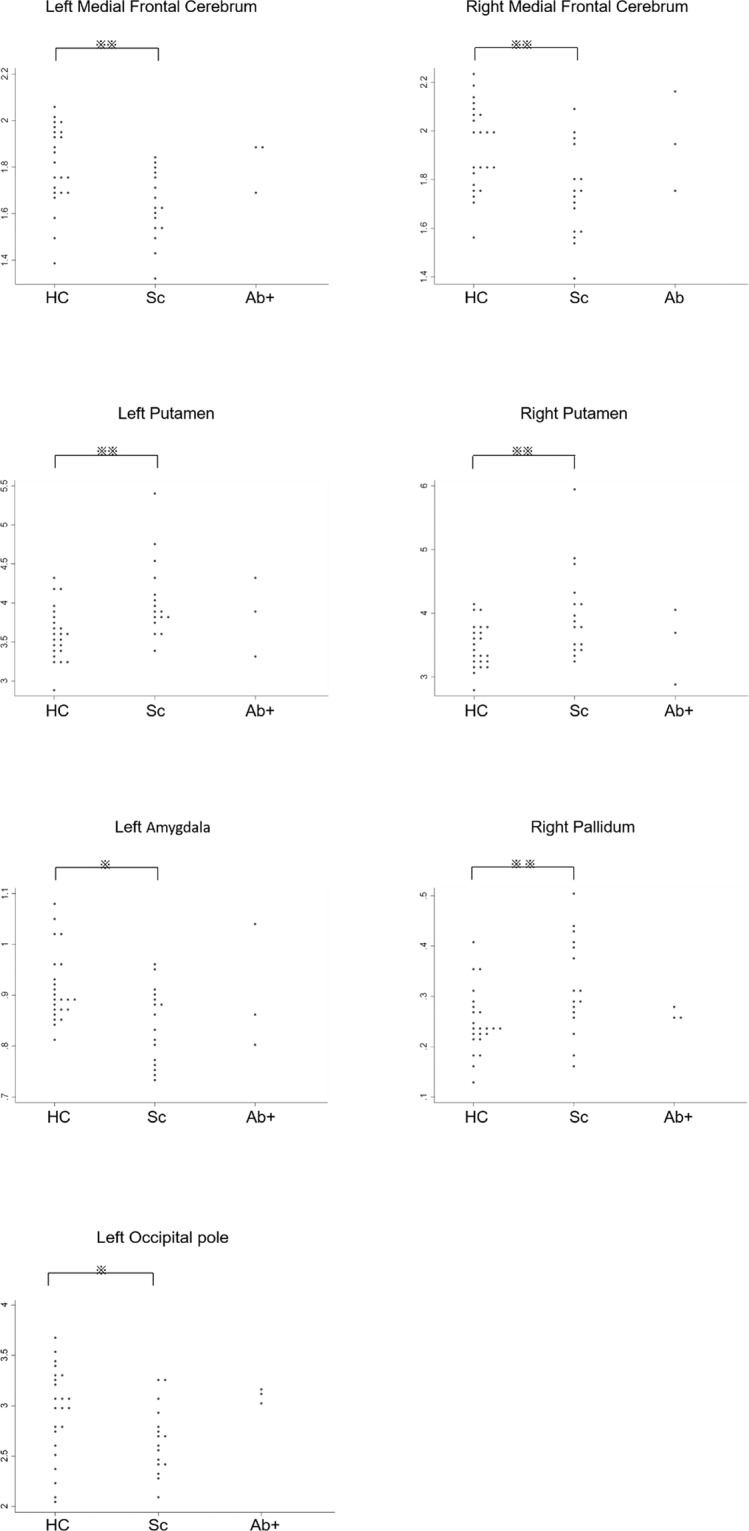
Significantly different brain volumes in antibody-negative patients with schizophrenia. Regional brain volumes are compared between healthy controls (HC), antibody-negative patients with schizophrenia (Sc), and antibody-positive patients with schizophrenia (Ab+). Only regions with statistical differences between HC and Sc are shown below. Significance was determined at p-values < 0.01 (**) and <0.05 (*) using the Kruskal–Wallis test followed by Dunn’s post hoc test.

Antibody-negative patients with schizophrenia showed compared to healthy controls increased volumes in the putamen (left, p = 0.003; right, p = 0.007) and right pallidum (p = 0.007), as well as decreased volumes in the left amygdala (p = 0.011), medial frontal cerebrum (left, p = 0.003; right, p = 0.004), and left occipital pole (p = 0.032; Fig. 5), which is compatible with previous reports^14–16^.

**Figure 6:**
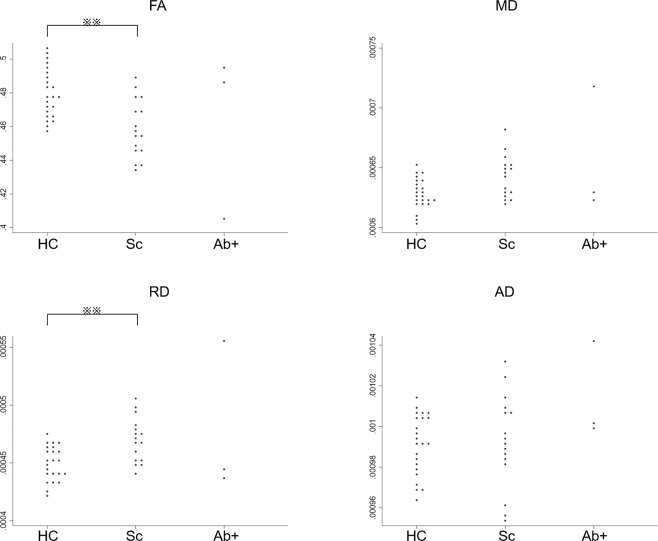
Diffusion parameters derived from diffusion tensor imaging. Fractional anisotropy (FA), mean diffusivity (MD), radial diffusivity (RD), and axial diffusivity (AD) values are compared between healthy controls (HC), antibody-negative patients with schizophrenia (Sc), and antibody-positive patients with schizophrenia (Ab+). Statistical significance was determined at p-value < 0.01 (**) using the Kruskal–Wallis test followed by Dunn’s post hoc test.

Diffusion MRI measurements (i.e., fractional anisotropy [FA], mean diffusivity [MD], radial diffusivity [RD], and axial diffusivity [AD]) revealed no significant features in antibody-positive patients (Fig. 6). By contrast, antibody-negative patients with schizophrenia showed decreased FA (p = 0.002) and increased RD (p = 0.003; Fig. 6) values compared to healthy controls in line with previous studies^17,18^.

The relationship between brain imaging characteristics and western blot reactivity in Fig. 3 (Sc4 > Sc6 > Sc5) was not obvious according to the Supplementary Figs. S3–S5, in which IDs (i.e., Sc4, Sc5, and Sc6) were labelled on the Figs. 4–6.

## Discussion

We isolated two proteins that reacted with pooled sera from patients with schizophrenia, but not with those from healthy controls, by using two-dimensional gel electrophoresis and western blotting. The proteins were identified by mass spectrometry as PDHA1 and DLAT. Both are components of the pyruvate dehydrogenase complex (PDH) in mitochondria. In our population, 3 out of 25 patients with schizophrenia presented immunoreactivity against the human recombinant PDHA1, whereas no participant showed an immunological reaction against human recombinant DLAT. The brain features of patients with schizophrenia whose sera reacted against PDHA1 differed from those without an immunological reaction. These findings suggest that the antibody targeting PDHA1, a component of the PDH in mitochondria, may be involved in the pathogenesis of a specific subgroup of schizophrenia.

The main function of mitochondria is to generate through oxidative phosphorylation energy for cells in the form of adenosine triphosphate (ATP). The brain uses more than 20% of the total body ATP, which is approximately 2% of the body mass^19^. As ATP is crucial to maintain and regulate the complex systems of the brain, mitochondrial dysfunction has been linked to several neuropsychiatric disorders such as Alzheimer’s disease, Parkinson’s disease, and autism^20–22^. Furthermore, the relationship between schizophrenia and mitochondrial dysfunction is supported by several lines of research. For instance, post-mortem studies have revealed structural and functional abnormalities in the mitochondria of patients with schizophrenia^23^. In addition, a genetic study has shown associations between nuclear-encoded mitochondrial variants and schizophrenia^24^. Thus, mitochondrial dysfunction is considered as one of the underlying pathophysiological mechanisms of schizophrenia.

PDH plays an important role in mitochondrial energy production, linking glycolysis to the tricarboxylic acid cycle and subsequent oxidative phosphorylation by catalysing the oxidative decarboxylation of pyruvate to generate acetyl-CoA^25^. The PDH complex is composed of enzymes including DLAT and PDHA1. Among the component enzymes, PDHA1 is a key to PDH functions. Alterations in PDHA1 and subsequent mitochondrial dysfunctions have been found in several neuropsychiatric disorders. For instance, accumulation of PDHA1 is reported in brainstem-type Lewy bodies of patients with idiopathic Parkinson’s disease and dementia with Lewy bodies^26^;these patients can develop psychotic symptoms. PDHA1 deficiency was first associated with schizophrenia in a report published in 2017^27^, which described a patient with a mitochondrial disease due to a mutation in the *PDHA1* gene who developed schizophrenia-like symptoms. These findings, along with our results, imply that antibodies targeting PDHA1, the key enzyme of mitochondrial energy production, may cause psychiatric symptoms in a specific subgroup of schizophrenia.

In the present study, MRI data revealed brain features in anti-PDHA1 antibody-positive patients that were different from those seen in ‘conventional’ schizophrenia. Typically, schizophrenia patients exhibit decreased volumes in the fusiform gyrus^28,29^. The fusiform gyrus has various neural functions related to recognition, such as face perception, object recognition, and reading^30^. A reduced volume of the fusiform gyrus, as well as the dysfunction of this brain region in schizophrenia, is considered as one of the pathophysiological mechanisms of impaired recognition, especially facial recognition^28,29^. However, in contrast to ‘conventional’ schizophrenia, the anti-PDHA1 antibody-positive patients showed increased volumes in the fusiform gyrus; this aberrant pattern in regional brain volumes was also evident in the cuneus. Increased volumes of the fusiform gyrus have been reported in individuals with synesthesia^31^. The involvement of the fusiform gyrus in synesthesia is supported by functional MRI and electroencephalography, in addition to brain anatomical, studies^32^. In synesthesia, the stimulation of a sensory modality triggers abnormal additional perceptions, which can result in hallucinations, or an abnormal perception in the absence of the corresponding external stimulus^33^. These findings suggest that people with synesthesia and the antibody-positive patients in our sample might share a common mechanism of hallucination. Further research is required to reveal the pathophysiology in the subgroup of schizophrenia with anti-PDHA1 antibodies.

The association between mitochondrial dysfunction and increased grey matter volume can be explained by the hypothesis of altered grey matter volume in autism. In healthy individuals, the grey matter volume decreases with age after reaching a maximum at 10 years of age^34^. In individuals with autism, early overgrowth, later slow growth, and subsequently increased grey matter volume have been reported^35,36^. This abnormal grey matter trajectory in autism is, at least in part, hypothesised as a result of oxidative stress^36^. Similar to autism, anti-PDHA1 antibody-positive cases can have excessive oxidative stress induced by mitochondrial dysfunction^37^ causing brain enlargement. Its trajectory – whether the observed brain enlargement is caused by developmental and/or ageing abnormalities – cannot be addressed due to our study design. Further studies in anti-PDHA1 antibody-positive cases to reveal molecular processes and longitudinal brain volume changes are required.

Regarding the identified DLAT protein, no patient serum showed an immunological reaction against human recombinant DLAT. This unexpected finding might be attributable to epitopic differences between the native protein found in two-dimensional gel electrophoresis and the human recombinant protein. For example, glycosylated DLAT may form an epitope. In this case, human recombinant DLAT without glycosylation would not be recognised by antibodies in the patient serum.

We should also note some limitations to be considered as follows: (1) The sample size was small and might not be representative, although brain MRI data in healthy controls and antibody-negative patients were compatible with those of previous reports. (2) The effects of the immune system in individual participants were not fully examined. For example, the wide spectrum of known autoantibodies^9^ was not evaluated in healthy controls. Besides, people with allergy were not excluded from the healthy control group. (3) The effects and mechanisms of anti-PDHA1 antibody on psychotic symptoms are undetermined. For example, whether the antibody is a cause or result of schizophrenia is unknown. (4) Cerebrospinal fluid (CSF) was not used in this study due to difficulties in sampling and preparation. The presence of the antibody in the CSF should be evaluated in future research. (5) To identify potential relationships as an exploratory analysis for a novel identified antibody, Bonferroni correction was not applied in the analysis of the brain data. Caution is needed in the interpretation of these results.

In this study, we detected anti-PDHA1 antibody in sera from a subgroup of patients with schizophrenia. Schizophrenia is considered a syndrome with various underlying mechanisms. The detected antibody could be useful in identifying a subgroup with specific schizophrenia pathophysiology.

## Methods

### Participants

We recruited 25 patients with schizophrenia who had been diagnosed with the patient edition of the Structured Clinical Interview for DSM-IV Axis I Disorders (SCID). Age- and sex-matched healthy participants for each of the 25 patients were also recruited. Healthy participants had no history of psychiatric diseases based on the non-patient edition of the SCID. Individuals with a history of head trauma, neurological disorder, substance abuse, or serious medical or surgical disorders such as malignant tumours and autoimmune disorders were excluded from this study. Participants were recruited through posters, word of mouth, direct approach, and an agency.

### Assessments and analyses of psychiatric symptoms

Symptom severity of schizophrenia was measured with the Positive and Negative Syndrome Scale (PANSS)^38^. The Global Assessment of Functioning scales both for symptoms (GAF-S) and functioning (GAF-F) were also used to evaluate the severity of symptoms and the level of functioning on a scale of 1 to 100 where higher scores indicate a better state^39^. In addition, the BACS was conducted to measure cognitive functions among patients with schizophrenia^40^. The premorbid IQ was estimated using the Japanese Adult Reading Test, which was utilised as an alternative to the National Adult Reading Test^41^. Quality of life in five domains (physical domain, psychological domain, social relationships, environment, and general health) was evaluated using the WHOQOL-26^42^.

Psychiatric measurements and demographic data were compared between healthy controls and patients with schizophrenia. Categorical data were analysed using Fisher’s exact test. Continuous data were analysed using the two-tailed independent sample t-test or the Mann–Whitney U test based on the results of the Shapiro–Wilk test. Statistical analyses were performed using IBM SPSS statistics 24.0.

### Antibody detection

1. Preparation of tissue proteins
  Two brains of Wister rats (*Rattus norvegicus*, 58 days old, one male and one female) were freeze-dried and powdered to extract and purify protein using either a “ProteoExtract” or a protein extraction kit (Calbiochem/EMD, Darmstadt, Germany) with the “2-D Clean-Up Kit” (GE Healthcare UK, Ltd., Buckinghamshire, England).
2. Two-dimensional polyacrylamide gel electrophoresis (PAGE)
  The purified protein of the rat brains was applied overnight to Immobiline Drystrip (p*I* 3–10, 18 cm; GE Healthcare)^43,44^. Then, the rehydrated strips were dried using a filter paper. Isoelectric focusing was performed in a Pharmacia Hoefer Multiphor II electrophoresis chamber (GE Healthcare). Subsequently, SDS-PAGE was performed in 9–18% acrylamide gradient gels in an IsoDalt electrophoresis chamber as the second dimension. The two-dimensional gels were stained with SYPRO Ruby (Invitrogen/Thermo Fisher Scientific, Waltham, MA, USA) protocols^45^. The SYPRO Ruby-stained protein spots were detected using a Molecular Imager FX (Bio-Rad Laboratories, Hercules, CA, USA). After imaging, all gels were also stained with silver and dried^46,47^.
3. Two-dimensional western blot analysis
  Proteins from the gels were subjected to a western blot analysis after being transferred to nitrocellulose membranes (Hybond ECL nitrocellulose membrane, RPN203D; Amersham/GE Healthcare)^48^. Two pools of sera obtained from 25 patients with schizophrenia and from 25 healthy controls, respectively, were prepared as a solution containing primary antibodies. The signals were detected by ECL Prime reagents and visualised on Hyperfilm ECL (GE Healthcare) using a peroxidase-labelled secondary antibody to human IgA + IgG + IgM (Code KPL5220-0331; Milford, MA, USA).
4. In-gel digestion and mass spectrometric identification of proteins
  Immunopositive protein spots were excised from the dried silver-stained two-dimensional gels and rehydrated for 20 min in 100 mM NH_4_HCO_3_. The gel spots were then destained for 20 min in a solution of 15 mM potassium ferricyanide and 50 mM thiosulfate, rinsed twice in ultrapure water, and finally dehydrated in 100% acetonitrile until they turned opaque white. The gel pieces were then dried in a vacuum centrifuge and subsequently rehydrated in a digestion solution consisting of 50 mM NH_4_HCO_3_ and 0.01 µg/µL modified sequence-grade trypsin (Promega, Madison, WI, USA). After overnight incubation at 37 °C, the digestion was terminated in 5% trifluoroacetic acid for 20 min. Peptides were extracted three times (20 min each) with 5% trifluoroacetic acid in 50% acetonitrile, and the extracted peptides were pooled and dried in a vacuum centrifuge^46,47^. The peptides were purified with ZipTip (Millipore, Burlington, MA, USA) according to the manufacturer’s protocol and analysed using an Ultraflextreme time-of-flight/time-of-flight (TOF/TOF; Bruker, Billerica, MA, USA) MALDI mass spectrometer and MASCOT database software (Matrix Science Inc., Boston, MA, USA).

### One-dimensional western blot analysis using human recombinant protein and individual sera

Based on the protein identification using mass spectrometry, we employed two human recombinant proteins. One was PDHA1 with an N-terminal His Tag (Aviva Systems Biology Corporation, USA; molecular weight, 42 kDa). The other was DLAT, whose cDNA is the coding component E2 of the multi-enzyme pyruvate dehydrogenase complex, fused to a hexahistidine purification tag (Prospec-Tany Technogene, Ltd., Israel; molecular weight, 60.63 kDa). These human recombinant proteins were subjected to one-dimensional gel electrophoresis using individual sera according to the following process. A total of 300 ng of each human recombinant protein was prepared by addition of dithiothreitol and NuPAGE LDS Sample Buffer, incubated at 70 °C for 10 min, and applied to wells of 4–12% Bis-Tris Protein Gels (Invitrogen/Thermo Fisher Scientific). PAGE was performed at 150 V for 45 min following the manufacturer’s instructions. The separated proteins were then transferred to polyvinylidene di-fluoride membranes (Hybond PVDF blotting membrane, 10600029; Amersham/GE Healthcare). The blots were reacted with individual sera as primary antibodies, which were detected by a peroxidase-labelled secondary antibody against human IgA + IgG + IgM (Code KPL5220-0331; Milford). Magic Mark XP Western Protein Standard molecular weight marker (Invitrogen/Thermo Fisher Scientific) was used for molecular weight determination. The presence or absence of a visible chemiluminescent signal at the same molecular size of the applied recombinant protein was used to define “positive” or “negative”, respectively.

### MRI acquisition and preprocessing

Patients with schizophrenia and healthy controls underwent MRI in a 3 T whole-body scanner equipped with a receiver-only 32-channel phased-array head coil (Tim Trio; Siemens, Erlangen, Germany). The scanning parameters of the T1-weighted 3-dimensional magnetisation-prepared rapid gradient-echo (3D-MPRAGE) sequence were as follows: repetition time, 2,000 ms; echo time, 3.4 ms; inversion time, 990 ms; field of view, 225 × 240 mm^2^; matrix, 240 × 256; slice thickness, 1.00 mm without gaps; number of slices, 208; and resolution, 0.9375 × 0.9375 × 1.0 mm^3^. The scanning parameters of the diffusion tensor imaging (DTI) sequence were as follows: repetition time, 5,640 ms; echo time, 106 ms; field of view, 192 × 192 mm^2^; imaging matrix, 96 × 96; 70 contiguous axial slices of 2 mm thickness. DTIs were acquired in 64 non-collinear directions of diffusion sensitisation (b = 1,500 s/mm^2^) and 5 non-diffusion-weighted images (b = 0 s/mm^2^).

To obtain individual regional grey matter volumes, the 3D-MPRAGE data were processed using the Computational Anatomy Toolbox 12 (CAT12; http://www.neuro.uni-jena.de/cat/) via the Statistical Parametric Mapping software 12 (SPM12; Wellcome Department of Imaging Neuroscience, London, UK) running in MATLAB software R2016b (The MathWorks, Inc., Natick, MA, USA). The CAT12 toolbox incorporated the following processes: (1) spatial registration to a reference brain (template), (2) tissue classification (segmentation) into grey and white matter and CSF, (3) bias correction of intensity non-uniformities, and (4) segmentation. The regional brain volume in each of the 142 regions of interest was obtained using maximum probability tissue labels derived from the Neuromorphometric atlas (Neuromorphometrics, Inc.; http://www.neuromorphometrics.com/). Obtained regional brain volumes were corrected for individual total intracranial volume, which was calculated as the sum of total grey matter, white matter, and CSF volumes. Adjusted regional brain volumes were used for further analyses.

DTI data were preprocessed as follows: (1) Denoising via the ‘dwidenoise’ command in MRtrix3 (http://mrtrix.readthedocs.io/en/latest/index.html) and (2) Subsequent preprocessing via dti_preprocess (https://github.com/RIKEN-BCIL/dti_preprocess) and FSL 6.0.1 (http://www.fmrib.ox.ac.uk/fsl) to correct eddy current distortion, head motion by registering each data point to the first b = 0 image with affine transformation, and image distortion caused by magnetic susceptibility artefacts by B0 unwarping. Subsequently, the FSL diffusion tensor fitting programme DTIFIT was run to produce maps of FA, MD, RD, and AD using the preprocessed data. The maps were analysed for diffusivity parameters using the Tract-Based Spatial Statistics tool (version 1.2) available in FSL^49^. This process is composed of the following steps: (1) A mean FA image was created by averaging all spatially normalised FA data using the FNIRT tool available in FSL^50^; (2) An original mean FA skeleton representing only the centres of white matter tracts common to all subjects was created by thinning the mean FA image; (3) A mean FA skeleton mask was generated with an FA threshold of 0.2 from the original mean FA skeleton; (4)The individual local maxima along the perpendicular direction of the voxel value in the normalised FA map were projected onto the mean FA skeleton; (5) Using the same projection vectors for FA, MD, RD, and AD, maps were projected onto the skeleton; and (6) The mean FA, MD, RD, and AD values were obtained based on the calculation within the skeleton.

### Analyses of brain imaging measures

Brain MRI data were analysed using the Kruskal–Wallis test followed by Dunn’s post hoc test to compare results between three groups (i.e., healthy controls, antibody-negative schizophrenia, and antibody-positive schizophrenia). The Kruskal–Wallis test, which requires at least three groups with a minimum sample size of three in each group, was selected^51^, whereas the one-way analysis of variance was not used due to the small sample size. A significance level of p < 0.05 was set for all statistical analyses on all available data using IBM SPSS statistics 24.0.

### Ethical approval

The study was conducted according to the tenets of the Declaration of Helsinki. Ethics approval was obtained from the Medical Ethics Committee of Kyoto University, Graduate School and Faculty of Medicine (R0027). All animal experiments were performed in accordance with institutional guidelines. All participants were aged over 18 years and provided written informed consent.

## Supplementary information

Supplementary Information.

## Data Availability

The data that support the findings of this study are available from the corresponding author, upon reasonable request.
