# Supplementary material for: Anti-PDHA1 antibody is detected in a subset of patients with schizophrenia
Source: Sci Rep. 2020 May 13;10:7906. doi: 10.1038/s41598-020-63776-0 (PMC7220915; doi:10.1038/s41598-020-63776-0)

Supplementary Information

Anti-PDHA1 antibody is detected in a subset of patients with schizophrenia

Authors:

Yukako Nakagami[1,2*], Genichi Sugihara[1], Noriyuki Nakashima[3], Masaaki Hazama[1], Shuraku Son[1], Shuhe Ma[4], Riki Matsumoto[5], Toshiya Murai[1], Akio Ikeda[6] & Kosaku Murakami[4]

[1] Department of Psychiatry, Kyoto University Graduate School of Medicine, Kyoto, Japan

[2] Kyoto University Health Service, Kyoto, Japan

[3] Department of Physiology, Kurume University School of Medicine, Kurume, Japan

[4] Department of Rheumatology and Clinical Immunology, Kyoto University Graduate School of Medicine, Kyoto, Japan

[5] Division of Neurology, Kobe University Graduate School of Medicine, Kobe, Japan

[6] Department of Epilepsy, Movement Disorders and Physiology, Kyoto University Graduate School of Medicine, Kyoto, Japan

***Corresponding author:**

Yukako Nakagami, MD, PhD,

Department of Psychiatry, Kyoto University Graduate School of Medicine

54 Shogoin-Kawahara-cho Sakyo-ku Kyoto 606-8507 Japan

TEL: +81-75-751-4947

FAX: +81-75-751-3382

Email: nakagami-kyt@umin.ac.jp

Supplementary Figure S1. Two-dimensional western blotting of rat brain proteins. Two-dimensional gel electrophoresis images other than those in Fig. 2 of the main text are shown. Two pools of sera from patients with schizophrenia (n=25, **a**) and healthy controls (n=25, **b**) were used at 1:6,400 dilution. As the secondary antibody, a peroxidase-labelled antibody to human IgA+IgG+IgM was used at 1:400,000 dilution.

Supplementary Figure S1a. As in Fig. 2a of the main text, the four arrows indicate the protein spots that reacted with pooled sera from 25 patients with schizophrenia. Among them, spot numbers 1 and 2 indicate proteins that reacted with the sera from patients with schizophrenia but not with those from healthy controls.

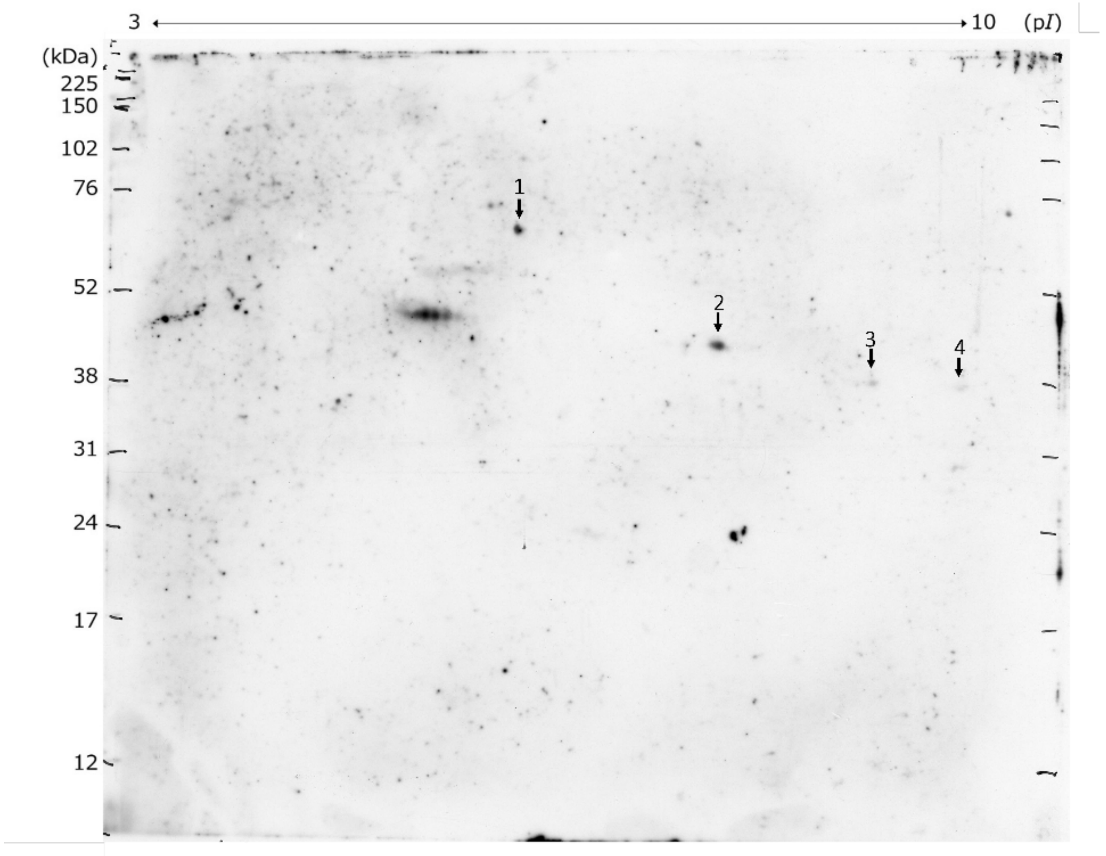

(200 μg rat brain protein was applied. kDa, kilodalton; p*I*, isoelectric point)

Supplementary Figure S1b. As in Fig. 2b of the main text, the two arrows (spot numbers 3’and 4’) indicate protein spots that reacted with pools of sera from patients with schizophrenia, as well as those from healthy controls.

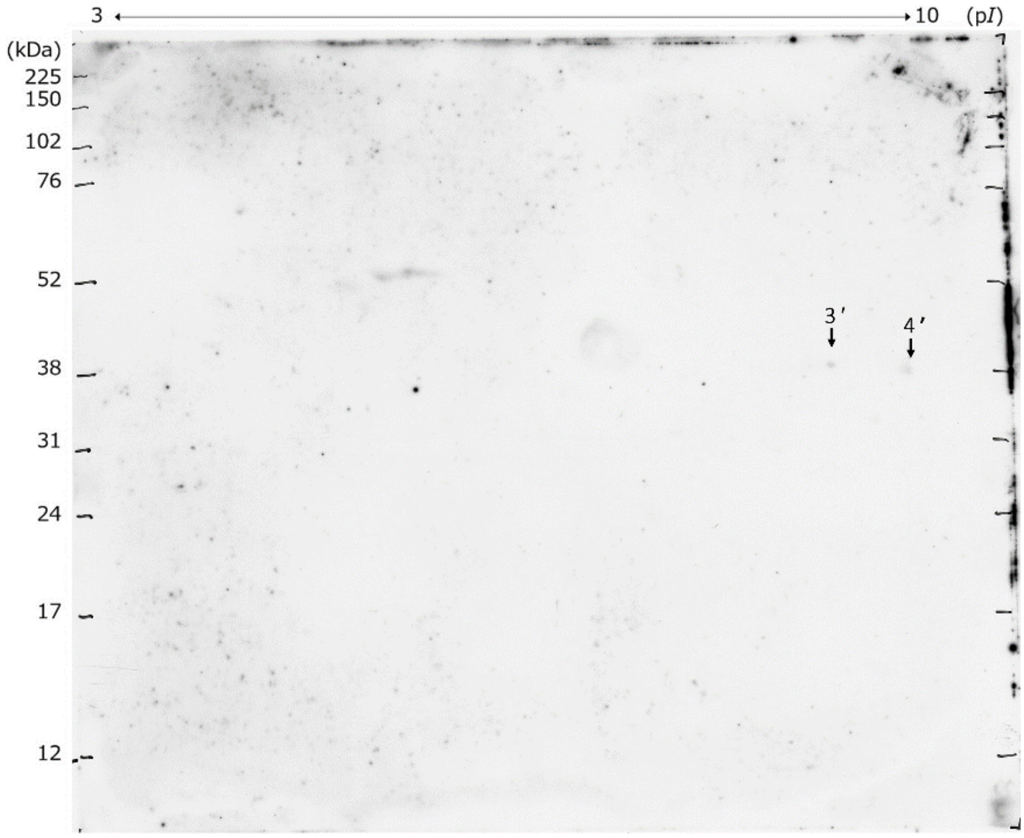

(100 μg rat brain protein was applied. kDa, kilodalton; p*I*, isoelectric point)

Supplementary Figure S2. Full original blots. Figures display the full original blots shown in Fig. 3 of the main text.

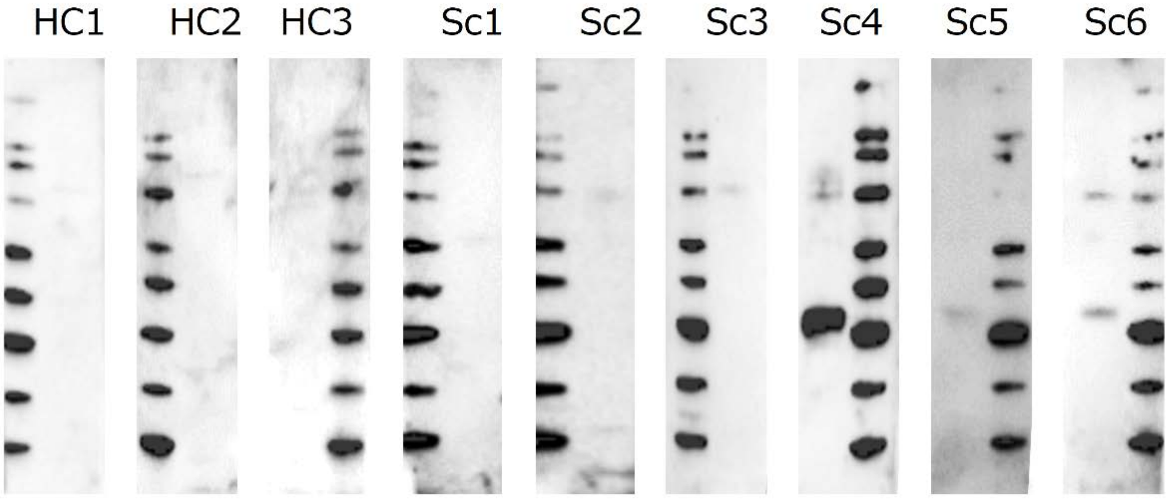

Supplementary Figure S3. The IDs of the antibody-positive cases in Fig. 3 (i.e., Sc4, Sc5, and Sc6) were added to Fig. 4 of the main text.

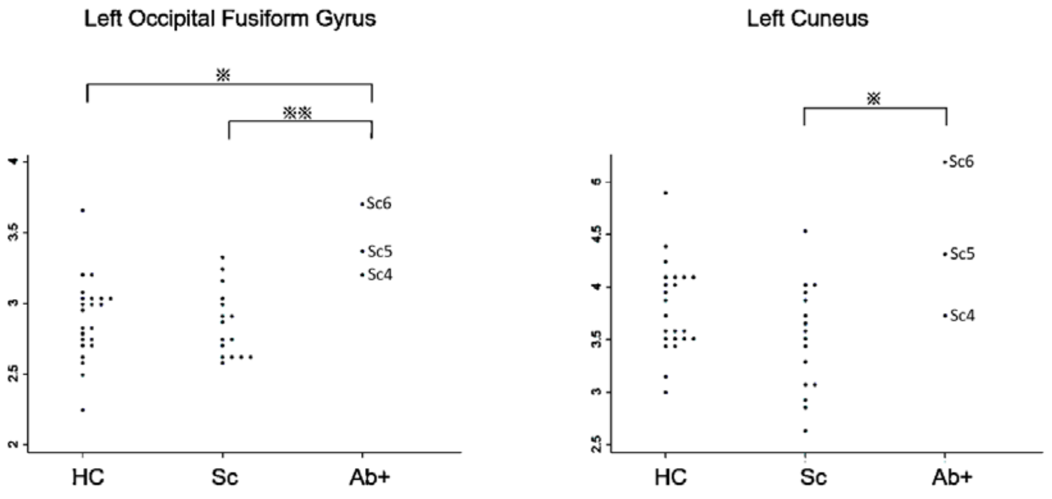

Supplementary Figure S4. The IDs of the antibody-positive cases in Fig. 3 (i.e., Sc4, Sc5, and Sc6) were added to Fig. 5 of the main text.

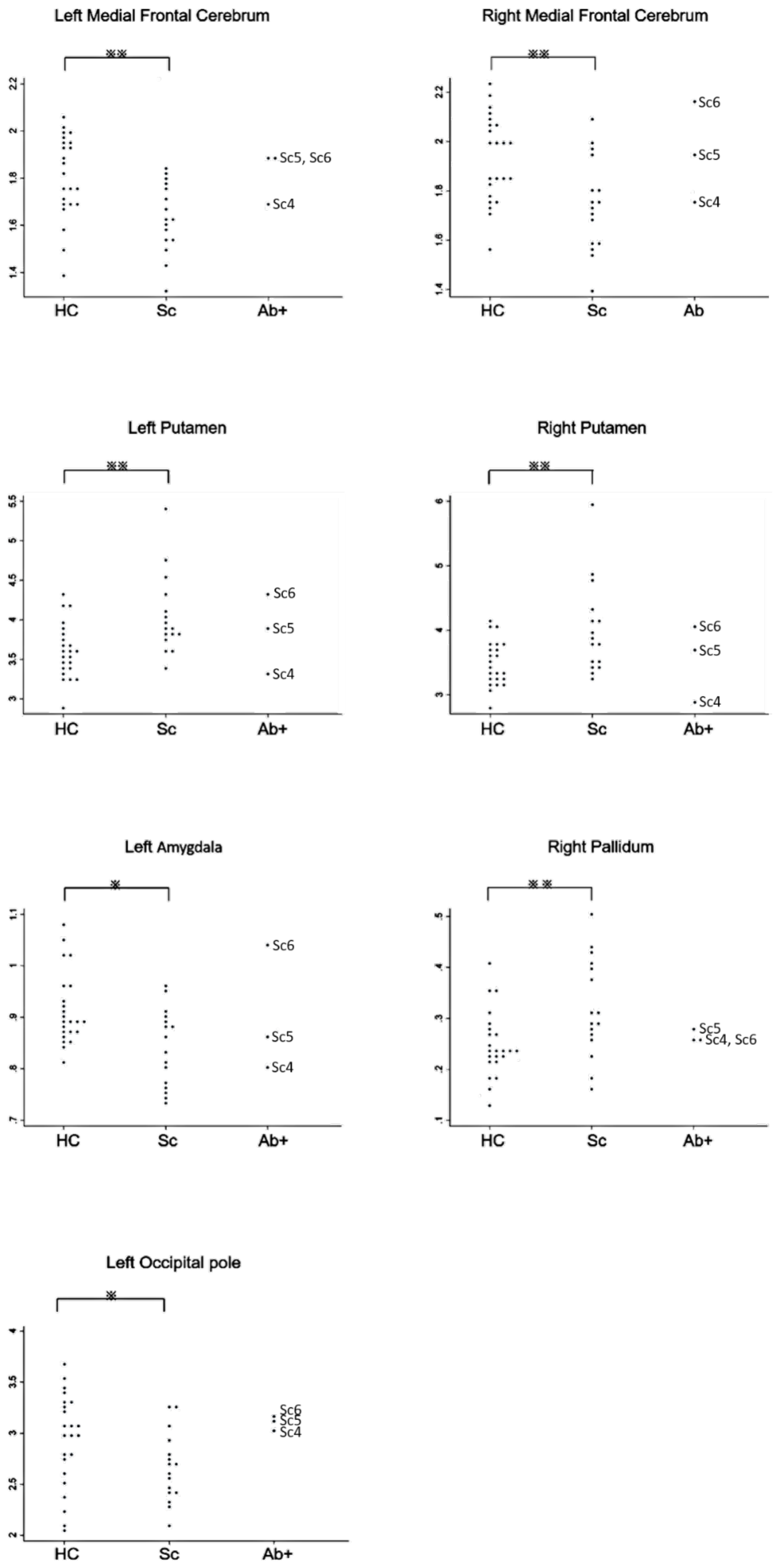

Supplementary Figure S5. The IDs of the antibody-positive cases in Fig. 3 (i.e., Sc4, Sc5, and Sc6) were added to Fig. 6 of the main text.

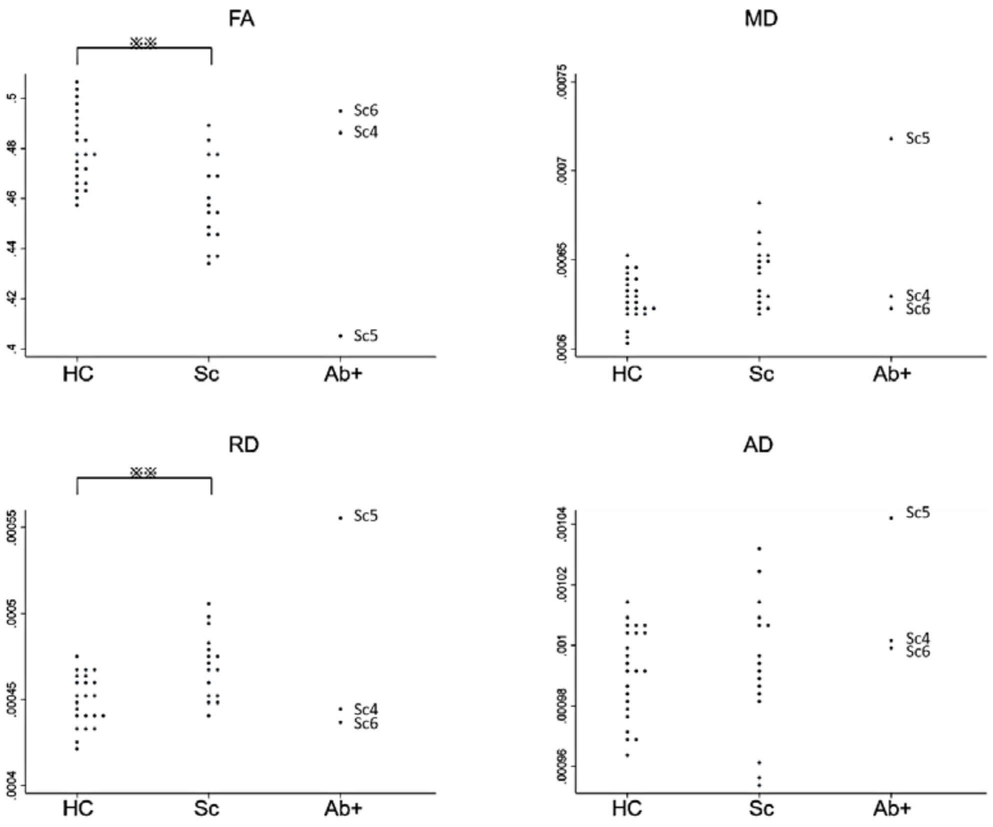

Supplement: Supplementary file 1 — Supplementary Information. [file 41598_2020_63776_MOESM1_ESM.pdf]
